# A model of calcium transport and regulation in the proximal tubule

**DOI:** 10.1152/ajprenal.00129.2018

**Published:** 2018-05-30

**Authors:** Aurélie Edwards, Olivier Bonny

**Affiliations:** ^1^Department of Biomedical Engineering, Boston University, Boston, Massachusetts; ^2^Department of Pharmacology and Toxicology, University of Lausanne, and Service of Nephrology, Lausanne University Hospital, Lausanne, Switzerland

**Keywords:** calcium, kidney, mathematical model, parathyroid hormone, phosphate

## Abstract

The objective of this study was to examine theoretically how Ca^2+^ reabsorption in the proximal tubule (PT) is modulated by Na^+^ and water fluxes, parathyroid hormone (PTH), Na^+^-glucose cotransporter (SGLT2) inhibitors, and acetazolamide. We expanded a previously published mathematical model of water and solute transport in the rat PT (Layton AT, Vallon V, Edwards A. *Am J Physiol Renal Physiol* 308: F1343–F1357, 2015) that did not include Ca^2+^. Our results indicate that Ca^2+^ reabsorption in the PT is primarily driven by the transepithelial Ca^2+^ concentration gradient that stems from water reabsorption, which is itself coupled to Na^+^ reabsorption. Simulated variations in permeability or transporter activity elicit opposite changes in paracellular and transcellular Ca^2+^ fluxes, whereas a simulated decrease in filtration rate lowers both fluxes. The model predicts that PTH-mediated inhibition of the apical Na^+^/H^+^ exchanger NHE3 reduces Na^+^ and Ca^2+^ transport to a similar extent. It also suggests that acetazolamide- and SGLT2 inhibitor-induced calciuria at least partly stems from reduced Ca^2+^ reabsorption in the PT. In addition, backleak of phosphate (PO_4_) across tight junctions is predicted to reduce net PO_4_ reabsorption by ~20% under normal conditions. When transcellular PO_4_ transport is substantially reduced by PTH, paracellular PO_4_ flux is reversed and contributes significantly to PO_4_ reabsorption. Furthermore, PTH is predicted to exert an indirect impact on PO_4_ reabsorption via its inhibitory action on NHE3. This model thus provides greater insight into the mechanisms that modulate Ca^2+^ and PO_4_ reabsorption in the PT.

## INTRODUCTION

Sixty to 70% of the filtered load of Ca^2+^ is reabsorbed in the proximal tubule (PT), mostly across the paracellular route, via passive diffusion and convection (i.e., solvent drag). There is some evidence (reviewed in Ref. 18) that a small fraction (10–20%) of Ca^2+^ is reabsorbed across the transcellular route, but the underlying molecular transporters remain to be elucidated. The respective contribution of diffusion and convection to Ca^2+^ fluxes in the PT and the extent to which the transport of Ca^2+^ is coupled to that of Na^+^ and water have yet to be fully understood (27, 39). The objective of the present study was to provide a quantitative description of the forces that drive Ca^2+^ reabsorption in the PT and to examine how Ca^2+^ transport in this segment is modulated by filtration rates, Na^+^, parathyroid hormone (PTH), acetazolamide, and inhibitors of Na^+^-glucose transporters (SGLTs). We expanded a model of water and solute transport in the PT that we published previously (26) but that did not include Ca^2+^.

We also modified the model’s handling of anionic inorganic phosphate (PO_4_). In plasma, PO_4_ is mostly present as ${\text{HPO}}_{4}^{2-}$ and ${\text{H}}_{\text{2}}{\text{PO}}_{\text{4}}^{-}$ in a 4:1 ratio. About 80% of the filtered load of PO_4_ is reabsorbed by the PT (27, 49). PO_4_ entry into the cell is mediated by three types of Na^+^-phosphate cotransporters: NaPi-IIa (SLC34A1), NaPi-IIc (SLC34A3), and PiT-2 (SLC20A2) (10). NaPi-IIa mediates cotransport of 3 Na^+^:1 ${\text{HPO}}_{4}^{2-}$, NaPi-IIc mediates cotransport of 2 Na^+^:1 ${\text{HPO}}_{4}^{2-}$, and PiT-2 mediates cotransport of 2 Na^+^:1 ${\text{H}}_{\text{2}}{\text{PO}}_{\text{4}}^{-}$. Whereas the previous version of the PT model only considered a generic apical 1 Na^+^:1 ${\text{H}}_{\text{2}}{\text{PO}}_{\text{4}}^{-}$ cotransporter, in the present study we accounted for these specific Na^+^-PO_4_ cotransporters. We also compared the direct and indirect ways in which PTH affects PO_4_ transport in the PT.

The PT model incorporates flow-dependent transport, i.e., the observation that high flow velocity augments transepithelial fluxes by increasing transporter membrane abundance (34). Du et al. (14) demonstrated that Na^+^ and $HCO_{3}^{-}$ reabsorption varies proportionally to the microvillous torque. Following the approach of Weinstein and colleagues (62), the abundance of transporters in apical and basolateral membranes is taken to be proportional to the torque. Flow-dependent transport plays an important role in maintaining perfusion-absorption balance. It may also act to prevent large excursions in the transepithelial fluxes of water and Na^+^ at a given perfusion rate.

## MODEL DESCRIPTION

### 

#### Conservation equations.

The mathematical model of transport along the PT of a male rat is based on conservation equations, which are solved at steady state. The PT consists of two cortical (S1–S2) segments (with a combined length taken as 0.97 cm) and a medullary (S3) segment (0.13 cm). We assume that all segments of the PT express the same types of channels, pumps, and cotransporters, with the exception of glucose transporters (26). Membrane surface areas are reduced by a factor of 2 in the S3 segment to account for decreased membrane infolding. As described above, luminal and peritubular transporter density increases linearly with the relative microvillous torque (62); the proportionality constant is set to 1.8 in the S1–S2 segment and to 0.9 in the S3 segment in the present study, so that the predicted reabsorption of Na^+^ and K^+^ equals approximately two-thirds of the filtered load.

**Fig. 1.**
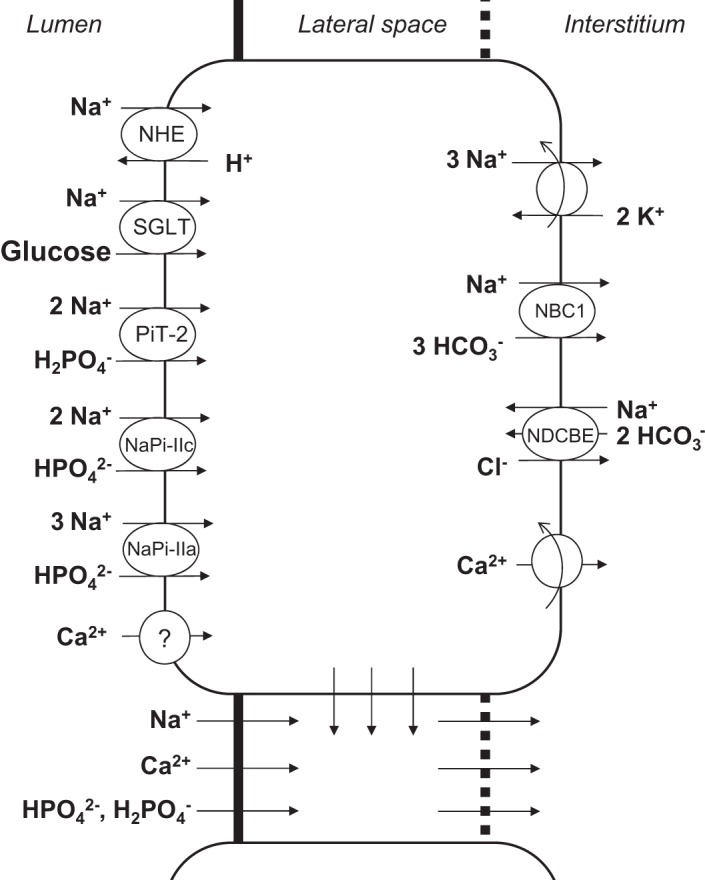
Model representation of a rat proximal tubule cell and the adjacent paracellular pathway. The model describes transport of water and 16 solutes. Only the main Na^+^ and Ca^2+^ transporters are shown. Ca^2+^ is assumed to enter the cell across an apical Ca^2+^ channel and exit via a plasma membrane Ca^2+^ pump. NHE, Na^+^/H^+^ exchanger; SGLT, Na^+^-glucose transporter; PiT-2, NaPi-IIc, and NaPi-IIa, Na^+^-PO_4_ transporters; NBC, Na^+^-$HCO_{3}^{-}$ cotransporter; NDCBE, Na^+^-dependent Cl^−^/$HCO_{3}^{-}$ exchanger.

As shown in Fig. 1, the model represents four compartments: the lumen (L), the cell cytosol (C), the lateral intercellular space (I), and the peritubular fluid (P). Conservation of Ca^2+^ in the lumen, cells, and intercellular space is written as follows:

(1) $$\frac{\text{d}\left({\text{F}}_{\text{V}}{\text{C}}_{\text{Ca}}^{\text{L}}\right)}{\text{d}x}={\text{S}}^{IL}J_{\text{Ca}}^{IL}+{\text{S}}^{CL}J_{\text{Ca}}^{CL}$$

(2) $$\begin{array}{cc} 0=\underset{\text{compartment}\;N}{\sum }{\text{S}}^{NM}J_{\text{Ca}}^{NM} & M=\text{C,}\;\text{ I} \end{array}$$

F_V_ represents the luminal flow rate, S*^NM^* is the surface area per unit length at the interface between compartments* N* and *M*, and *J^NM^* is the flux across that interface. Note that at steady state the net flux of Ca^2+^ from intracellular stores to the cytosol and the net rate of Ca^2+^ binding to Ca^2+^ buffers in the cytosol and the sarcoplasmic reticulum are zero (for their dynamic expressions see Ref. 12).

#### Ca^2+^ fluxes.

The Ca^2+^ flux from the lumen to compartment* M* (*M* = C, I) is computed as follows:

(3a) $$J_{\text{Ca}}^{\text{L}M}=J_{\text{V}}^{\text{L}M}\left(1-\sigma_{\text{Ca}}^{\text{L}M}\right){\overset{¯}{\text{C}}}_{\text{Ca}}+P_{\text{Ca}}^{\text{L}M}\zeta_{\text{Ca}}^{\text{L}M}\left(\frac{{\text{C}}_{\text{Ca}}^{\text{L}}-{\text{C}}_{\text{Ca}}^{M}\exp(-\zeta_{\text{Ca}})}{1-\exp\left(-\zeta_{\text{Ca}}^{LM}\right)}\right)$$

(3b) $${\bar{\text{C}}}_{\text{Ca}}=\frac{{\text{C}}_{\text{Ca}}^{\text{L}}-{\text{C}}_{\text{Ca}}^{M}}{\ln{\text{C}}_{\text{Ca}}^{\text{L}}-\ln{\text{C}}_{\text{Ca}}^{M}}$$

(3c) $$\zeta_{\text{Ca}}^{\text{L}M}=\frac{z_{\text{Ca}}F}{RT}\left(\Psi^{\text{L}}-\Psi^{M}\right)$$

The first term in *Eq. 3*a corresponds to the convective component of the flux: *J*_V_ is the volume flux (superscripts are omitted for simplicity), ${\bar{\text{C}}}_{\text{Ca}}$ is a (logarithmic) mean concentration, and σ_Ca_ is the reflection coefficient of the membrane to Ca^2+^; σ_Ca_ is set to 1 for cell membranes, 0.89 for the tight junction (TJ) (41), and 0 for the basement membrane (BM; at the interface between the lateral interspace and the peritubular fluid). The second term corresponds to the electrodiffusive component: *P*_Ca_ is the membrane permeability to Ca^2+^, and ζ_Ca_ is a normalized electric potential difference, a function of the valence *z*_Ca_ of Ca^2+^ and the electric potential Ψ. *R* and *F* are the ideal gas and Faraday constants, respectively.

The permeability of the TJ to Ca^2+^ is taken as 20 × 10^−5^ cm/s based on the compilation in Ref. 18. The permeability of the BM to Ca^2+^ is computed based on the permeability to Na^+^, given a Ca^2+^-to-Na^+^ free diffusivity ratio of 7.93:13.3 (30). The permeability of the apical membrane to Ca^2+^ is set to 0.005 × 10^−5^ cm/s so as to yield a transcellular flux amounting to 15% of the overall flux.

The molecular mechanisms by which Ca^2+^ is extruded from PT cells remain to be identified. Based on recent transcriptomic data (28), we neglect the potential contribution of Na^+^/Ca^2+^ exchangers and assume that Ca^2+^ is pumped out of the cell by plasma membrane Ca^2+^-ATPase (PMCA) at rate given by

(4) $$J_{\text{Ca}}^{\text{PMCA}}=J_{\max}^{\text{PMCA}}\;\left(\frac{{\text{C}}_{\text{Ca}}^{C}}{{\text{C}}_{\text{Ca}}^{C}+K_{\text{m}}^{\text{PMCA}}}\right)$$

The affinity of the pump to Ca^2+^ ($K_{\text{m}}^{\text{PMCA}}$) is taken as 75.6 nM (57) and the maximum flux ($J_{\text{max}}^{\text{PMCA}}$) as 0.5 × 10^−9^ mmol·cm^−2^·s^−1^. Parameter values are summarized in Table 1.

**Table 1.** Ca^2+^ and PO_4_ parameter values

Parameter	Value	Reference
Permeability to Ca^2+^, cm/s		
Tight junction	20 × 10^−5^	18
Basement membrane	60 × 10^−5^	Based on 30
Apical cell membrane	0.005 × 10^−5^	Adjusted
PMCA maximum flux, mmol·cm^−2^·s^−1^	0.5 × 10^−9^	Adjusted
PMCA affinity to Ca^2+^, nM	75.6	57
Reflection coefficient of tight junction to Ca^2+^	0.89	41
Transporter density, mmol^2^·J^−1^·s^−1^·cm^−2^		
NaPi-IIa	0.30 × 10^−9^	Adjusted
NaPi-IIc	0.25 × 10^−9^ (0 in S3)	Adjusted
PiT-2	0.10 × 10^−9^ (0 in S3)	Adjusted

PMCA, plasma membrane Ca^2+^-ATPase; S3, medullary segment of the proximal tubule.

#### Interstitial concentration gradient.

Interstitial fluid concentrations are prescribed in this model: they are equal to plasma concentrations in the cortex and increase linearly along the corticomedullary axis in the medulla. Based on our macroscopic model of Ca^2+^ transport in different populations of nephrons, vasa recta, and the interstitium (56), which did not represent processes at the cell/molecular level, we assume that the interstitial Ca^2+^ concentration ([Ca^2+^]) increases linearly from 1.25 mM at the corticomedullary junction to 1.60 mM at the end of the S3 segment and to 2.50 mM at the junction between the outer medulla (OM) and inner medulla (IM). The single-nephron glomerular filtration rate (SNGFR) is taken as 30 nl/min, and the filtered load of Ca^2+^ is 37.5 pmol/min per nephron.

#### PO_4_ transport.

In previously published models of the PT (26, 62), the apical PO_4_ transporter was represented as a 1 Na^+^:1 ${\text{H}}_{\text{2}}{\text{PO}}_{\text{4}}^{-}$ cotransporter. It is now known that PO_4_ entry into PT cells is mediated by the cotransporters NaPi-IIa, NaPi-IIc, and PiT-2 (11, 27); their respective stoichiometries are as follows: 3 Na^+^:1 ${\text{HPO}}_{4}^{2-}$, 2 Na^+^:1 ${\text{HPO}}_{4}^{2-}$, and 2 Na^+^:1 ${\text{H}}_{\text{2}}{\text{PO}}_{\text{4}}^{-}$. Hence, NaPi-IIa and PiT-2 are electrogenic, whereas NaPi-IIc is electroneutral. Fluxes across these cotransporters are computed using the nonequilibrium thermodynamic approach (60). Based on the immunochemistry findings of Picard et al. (42), we assume that NaPi-IIa is expressed along the full PT and that NaPi-IIc and PiT-2 are only present in the convoluted PT. The transport coefficient (a measure of density) of NaPi-IIa is set to 0.30 × 10^−9^ mmol^2^·J^−1^·s^−1^·cm^−2^ everywhere, and those of NaPi-IIc and PiT-2 are taken as 0.25 × 10^−9^ and 0.10 × 10^−9^ mmol^2^·J^−1^·s^−1^·cm^−2^, respectively, in the S1–S2 segment and zero in the S3 segment. No other PO_4_ cellular entry pathways are considered. The interstitial concentration of PO_4_ is taken to increase from 2.6 mM at the corticomedullary junction to 3.9 mM at the OM-IM junction (58), and the filtered load of PO_4_ is 78.0 pmol/min per nephron.

## RESULTS

### 

#### Forces driving Ca^2+^ reabsorption.

**Fig. 2.**
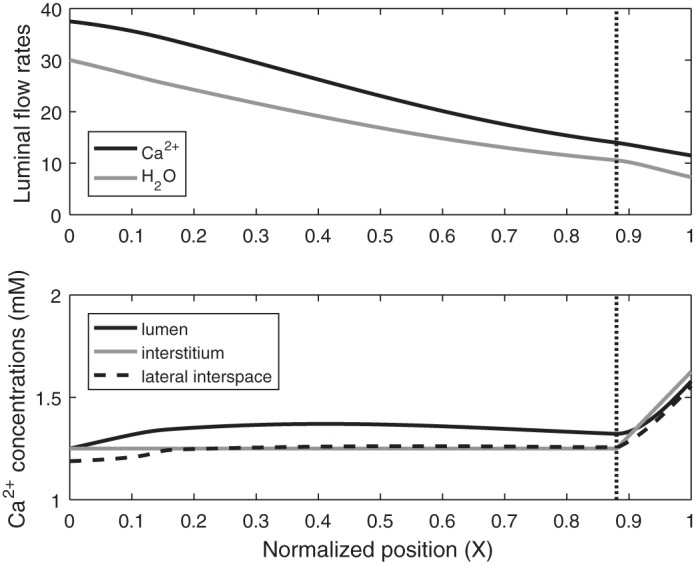
Base-case Ca^2+^ flows and concentrations along the proximal tubule. *Top*: molar flow rate of Ca^2+^ (in pmol/min per nephron) and H_2_O (in nl/min per nephron) in the lumen as a function of normalized position X (i.e., position divided by proximal tubule length). *Bottom*: Ca^2+^ concentration in lumen, interstitium, and lateral interspace as a function of X. Vertical line denotes the boundary between the cortex and the medulla, which marks the transition between the S2 and S3 segments.

Under baseline conditions, the model predicts that the PT reabsorbs 68–70% of the filtered load of Na^+^, K^+^, and Cl^−^. Fractional Ca^2+^ reabsorption is 69.3%, and the tubular fluid-to-glomerular filtrate [Ca^2+^] ratio [(TF/GF)_Ca_] increases from 1.0 to 1.3, in accordance with reported measurements (reviewed in Ref. 18). As depicted in Fig. 2, the molar flow rate of Ca^2+^ in the lumen decreases in parallel with volume flow. As the rate of water reabsorption increases significantly in the medullary (S3) segment, owing to the interstitial osmolality gradient, so do [Ca^2+^] in the lumen and (TF/GF)_Ca_ (Fig. 2).

**Fig. 3.**
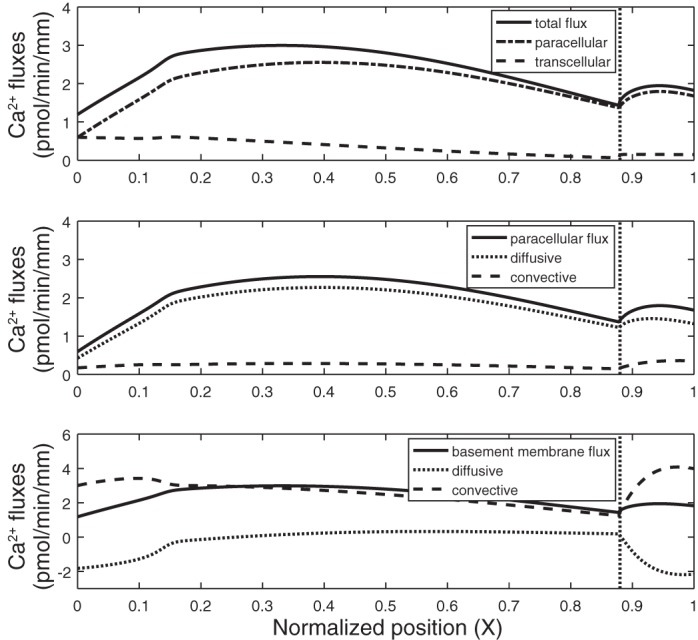
Ca^2+^ fluxes along the proximal tubule in the base case. *Top*: total transepithelial Ca^2+^ flux is the sum of Ca^2+^ fluxes across the tight junction (paracellular) and the apical cell membrane (transcellular). *Middle*: paracellular Ca^2+^ flux is the sum of its electrodiffusive and convective components. *Bottom*: Ca^2+^ flux across the basement membrane is mostly driven by convection. At steady state, it is the sum of the tight junction flux and flux from the lateral cell membrane into the lateral interspace. Length-averaged values are given in Table 1. Vertical line denotes the boundary between the cortex and the medulla.

Results are given on a per-nephron basis: *J*_Ca_ denotes the local or average Ca^2+^ flux (in pmol·min^−1^·mm^−1^) and *T*_Ca_ denotes the rate of Ca^2+^ reabsorption along the entire PT (in pmol/min). The base-case *T*_Ca_ is computed as 26.0 pmol/min, 85% of which is paracellular. Equivalently, the average *J*_Ca_ equals 2.36 pmol·min^−1^·mm^−1^ (Table 2). Paracellular transport of Ca^2+^ across the TJ, which separates the lumen from the lateral interspace, is followed by Ca^2+^ transport across the BM, which separates the lateral interspace from the interstitium (Fig. 1). Across the TJ, the Ca^2+^ flux is predominantly governed by electrodiffusion, rather than by convection (i.e., solvent drag). As water reabsorption raises [Ca^2+^] in the lumen above that in the lateral interspace and the interstitium (Fig. 2), the [Ca^2+^] gradient across the TJ drives Ca^2+^ reabsorption (Fig. 3). Since the reflection coefficient of the TJ to Ca^2+^ is 0.89 (41), i.e., close to 1, solvent drag across this barrier is limited (see *Eq. 3*a).

**Table 2.** Predicted Ca^2+^ fluxes and fractional reabsorption

	*J*_Ca_, pmol·min^−1^·mm^−1^ per nephron			
Total	Paracellular	Transcellular	%Reabsorption
Base case	2.36	2.02 (1.77 + 0.25)	0.34	69.3
5-fold decrease in$P_{\text{Ca}}^{\text{ap}}$	2.35	2.28 (2.03 + 0.25)	0.07	69.0
5-fold increase in $P_{\text{Ca}}^{\text{ap}}$	2.41	0.86 (0.62 + 0.24)	1.55	70.9
2-fold decrease in $P_{\text{Ca}}^{\text{tj}}$	2.20	1.84 (1.58 + 0.26)	0.36	64.8
10-fold decrease in$P_{\text{Ca}}^{\text{tj}}$	1.40	0.99 (0.70 + 0.29)	0.41	41.2
2-fold increase in $P_{\text{Ca}}^{\text{tj}}$	2.42	2.10 (1.86 + 0.24)	0.32	71.0
10-fold increase in$P_{\text{Ca}}^{\text{tj}}$	2.46	2.16 (1.92 + 0.24)	0.30	72.1
10-fold decrease in$P_{\text{Ca}}^{\text{bm}}$	2.44	2.11 (1.87 + 0.24)	0.33	71.7
10-fold increase in $P_{\text{Ca}}^{\text{bm}}$	2.33	1.99 (1.74 + 0.25)	0.34	68.5
$\sigma_{\text{Ca}}^{\text{tj}}$ = 0	2.54	2.24 (0.09 + 2.15)	0.30	74.6
$\sigma_{\text{Ca}}^{\text{tj}}$ = 1	2.33	1.99 (1.99 + 0)	0.34	68.6
Interstitial [Ca^2+^] at the OM-IM junction = 3.75 mM	2.27	1.93 (1.67 + 0.26)	0.34	66.5
Interstitial [Ca^2+^] at the OM-IM junction = 2.0 mM	2.40	2.06 (1.81 + 0.25)	0.34	70.4
40% increase in NHE3 and NBC1 expression and 10% increase in Na^+^-K^+^-ATPase expression	2.48	2.21 (1.94 + 0.27)	0.27	72.8
CA inhibition in the PT lumen	1.82	1.34 (1.17 + 0.17)	0.48	53.3
90% inhibition of NBC1	1.79	1.21 (1.03 + 0.18)	0.58	52.6
100% inhibition of SGLT2	2.07	1.66 (1.45 + 0.21)	0.41	60.8
100% inhibition of SGLT2 + 15% decrease in SNGFR	1.64	1.34 (1.17 + 0.17)	0.30	56.6

Values in parentheses show decomposition of Ca^2+^ flux (*J*_Ca_) into its diffusive and convective components, respectively. $P_{\text{Ca}}^{\text{ap}}$, $P_{\text{Ca}}^{\text{tj}}$, and $P_{\text{Ca}}^{\text{bm}}$, permeability of apical cell membrane, tight junction, and basement membrane to Ca^2+^; $\sigma_{\text{Ca}}^{\text{tj}}$, reflection coefficient of the tight junction to Ca^2+^; [Ca^2+^], Ca^2+^ concentration; OM-IM, outer medulla-inner medulla; CA, carbonic anhydrase; NBC1, basolateral Na^+^-$HCO_{3}^{-}$ cotransporter; SGLT2, Na^+^-glucose cotransporter type 2; SNGFR, single-nephron glomerular filtration rate.

At steady state, the flux of Ca^2+^ across the BM is the sum of the Ca^2+^ flux across the TJ and the Ca^2+^ flux from the lateral membrane of cells into the lateral interspace. As shown in Fig. 3, Ca^2+^ transport across the BM is primarily driven by convection. Solvent drag is significantly enhanced, relative to the TJ, because the reflection coefficient of the BM to Ca^2+^ (and all other electrolytes) is zero; moreover, there is significant water reabsorption from the cell into the lateral interspace, so that the water flux across the BM is larger than that across the TJ. The rapid convective transport of Ca^2+^ into the interstitium lowers [Ca^2+^] in the lateral interspace below that in the interstitium along parts of the tubule (Fig. 2), thereby eliciting backdiffusion of Ca^2+^ across the BM (Fig. 3).

Note that electrodiffusion is governed by both the transmembrane [Ca^2+^] gradient and the transmembrane voltage (ΔΨ). The electric potential in the lumen is predicted to rise from −0.22 mV at the PT inlet to a maximum of 1.31 mV about halfway through the tubule and to decrease to +0.94 mV at the outlet. The electric potential in the lateral interspace remains between −0.06 and +0.01 mV in the cortex and the medulla. As ΔΨ across the TJ changes sign and becomes lumen-positive, its contribution to the electrodiffusive flux increases. Nevertheless, our computations suggest that, overall, ΔΨ exerts a lower driving force than the lumen-to-lateral interspace [Ca^2+^] gradient.

In the renal medulla, the axial interstitial osmolarity gradient accelerates the rate of water removal from the PT lumen, thereby augmenting convective Ca^2+^ fluxes in the S3 segment (Fig. 3). The large increase in solvent drag across the BM is partly counteracted by enhanced Ca^2+^ diffusion in the opposite direction, as the lateral interspace [Ca^2+^] increasingly lags behind the interstitial [Ca^2+^] (Fig. 2).

#### Impact of transcellular Ca^2+^ permeability.

**Fig. 4.**
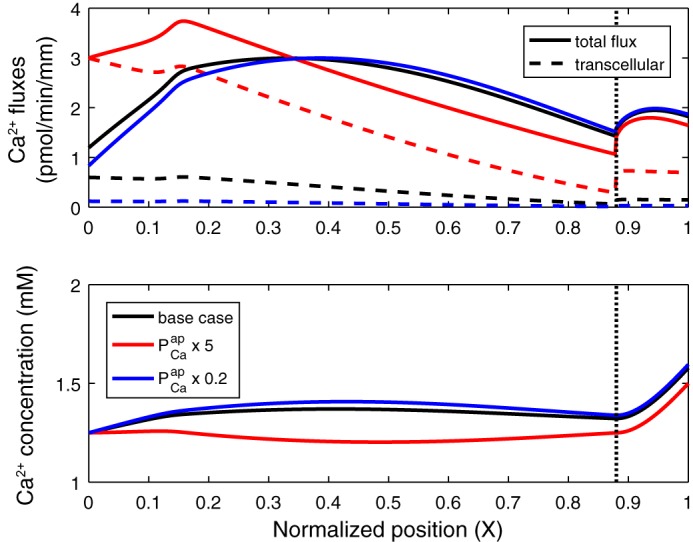
Impact of permeability of the apical membrane to Ca^2+^ ($P_{\text{Ca}}^{\text{ap}}$) on Ca^2+^ fluxes and concentrations along the proximal tubule. *Top*: total and transcellular Ca^2+^ fluxes. Paracellular flux is the difference between the 2 curves. *Bottom*: luminal Ca^2+^ concentration. $P_{\text{Ca}}^{\text{ap}}$ is set to its baseline value (base case), increased by a factor of 5 (× 5), or decreased by a factor of 5 (× 0.2). A 10-fold increase in $P_{\text{Ca}}^{\text{ap}}$ is predicted to result in Ca^2+^ secretion via the paracellular route (not shown). Vertical line denotes the boundary between the cortex and the medulla.

The contribution of transcellular Ca^2+^ transport in the PT and its underlying mechanisms are not understood. We chose the baseline value of the apical membrane Ca^2+^ permeability ($P_{\text{Ca}}^{\text{ap}}$) so that transcellular *T*_Ca_ represents 15% of total *T*_Ca_. The impact of varying $P_{\text{Ca}}^{\text{ap}}$ is illustrated in Fig. 4. The maximum PMCA flux was varied by the same factor as $P_{\text{Ca}}^{\text{ap}}$ to maintain intracellular [Ca^2+^] at ∼100 nM. The model predicts that increasing $P_{\text{Ca}}^{\text{ap}}$ and, thus, the transcellular *J*_Ca_ conversely reduces paracellular *J*_Ca_, because it accelerates Ca^2+^ removal from the lumen, thereby diminishing the lumen-to-interstitium [Ca^2+^] gradient. In fact, the paracellular *J*_Ca_ decreases to the extent that even though the net *J*_Ca_ is higher than in the base case in the early part of the PT, it is lower in the late PT. Conversely, decreasing the transcellular *J*_Ca_ augments the paracellular *J*_Ca_, as the lumen-to-interstitium [Ca^2+^] gradient becomes larger. Consequently, overall Ca^2+^ reabsorption is not much affected by variations in $P_{\text{Ca}}^{\text{ap}}$ (Table 2).

#### Impact of solvent drag.

**Fig. 5.**
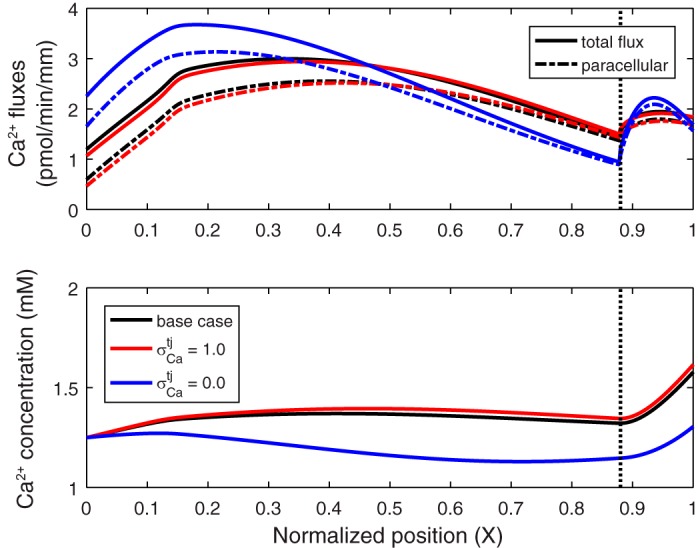
Ca^2+^ fluxes and concentrations along the proximal tubule for different values of the reflection coefficient of the tight junction to Ca^2+^ ($\sigma_{\text{Ca}}^{\text{tj}}$): 0.89 (base case) 1.0, and 0.0. *Top*: total and paracellular Ca^2+^ fluxes. *Bottom*: luminal Ca^2+^ concentration. Vertical line denotes the boundary between the cortex and the medulla.

We examined the effects of solvent drag on *J*_Ca_ by varying the reflection coefficient of the TJ to Ca^2+^ ($\sigma_{\text{Ca}}^{\text{tj}}$) from 0 to 1. Decreasing $\sigma_{\text{Ca}}^{\text{tj}}$ from its baseline value of 0.89 to 0, that is, enhancing the convective transport of Ca^2+^ across the TJ (see *Eq. 3a*), is predicted to substantially increase the paracellular *J*_Ca_ in the first half of the PT, relative to the base case, and to lower it in the second half due to the resulting decrease in the lumen-to-interstitium [Ca^2+^] gradient (Fig. 5). Because of these counterbalancing effects, overall Ca^2+^ reabsorption is only 7.6% higher (28.0 pmol·min^−1^·mm^−1^) than in the base case. Conversely, increasing $\sigma_{\text{Ca}}^{\text{tj}}$ from 0.89 to 1.0 slightly reduces (by 1.1%) overall Ca^2+^ reabsorption.

#### Impact of BM permeability.

Permeability of the BM to Ca^2+^ ($P_{\text{Ca}}^{\text{bm}}$) has not been measured. As shown in Table 2, the model predicts that a 10-fold decrease in $P_{\text{Ca}}^{\text{bm}}$ raises Ca^2+^ reabsorption by 3.4%, because it reduces the backdiffusion of Ca^2+^ across the BM. A 10-fold increase in $P_{\text{Ca}}^{\text{bm}}$ has opposite, albeit smaller, effects: it lowers Ca^2+^ reabsorption by 1.2%.

#### Impact of axial interstitial gradient.

Another uncertainty relates to the interstitial axial [Ca^2+^] gradient in the medulla. In the base case, interstitial [Ca^2+^] is taken to double between the cortex and the OM-IM junction. If we assume, instead, a threefold increase, the lag between luminal [Ca^2+^] and interstitial [Ca^2+^] in the medulla increases (results not shown). Hence, paracellular Ca^2+^ reabsorption is reduced along the S3 segment, from 2.51 pmol/min in the base case to 1.46 pmol/min, and overall Ca^2+^ reabsorption decreases from 26.0 to 24.9 pmol/min. Conversely, a smaller medullary [Ca^2+^] gradient is predicted to enhance the diffusion of Ca^2+^ across the paracellular pathway in the S3 segment and augment its overall reabsorption (Table 2).

#### Effects of glomerular filtration rate variations.

To examine the coupling between the transport of Ca^2+^ and that of water and/or Na^+^, we first simulated a 20% increase in the SNGFR. As described above, the model accounts for flow-dependent transport (14, 34, 62). A high flow rate in the PT lumen acts via the microvillous torque to recruit more transporters to the cell membrane, thereby enhancing transcellular fluxes and maintaining perfusion-absorption balance. The model thus predicts that a 20% increase in the filtered load of fluid and solutes results in substantially higher absolute reabsorption rates, but the fractional reabsorption of water, Na^+^, and Ca^2+^ increases only by 3–4% (from 69.3% to 73.1% for Ca^2+^). More specifically, as the filtered load of Ca^2+^ is raised from 37.5 to 45.0 pmol/min per nephron, *T*_Ca_ increases from 26.0 to 32.9 pmol/min, and the length-averaged *J*_Ca_ increases from 2.36 to 2.99 pmol·min^−1^·mm^−1^. Ca^2+^ transport is elevated across both pathways: transcellular *J*_Ca_ increases owing to enhanced membrane abundance of Ca^2+^ transporters, and paracellular *J*_Ca_ increases because the higher rate of water reabsorption augments the lumen-to-interstitium [Ca^2+^] gradient.

Conversely a 20% decrease in the SNGFR reduces the microvillous torque in the PT lumen, subsequently lowering transporter abundance at the membrane and absolute reabsorption rates. The fractional reabsorption of water, Na^+^, and Ca^2+^ is predicted to then decrease by 5–7% (from 69.3% to 62.7% for Ca^2+^). With a Ca^2+^ filtered load of 30.0 pmol/min per nephron, *T*_Ca_ equals 18.8 pmol/min, and the length-averaged *J*_Ca_ equals 1.71 pmol·min^−1^·mm^−1^ (Table 3). Ca^2+^ transport is reduced both across cells, due to fewer Ca^2+^ transporters at the membrane, and between cells, due to the lower lumen-to-interstitium [Ca^2+^] gradient.

**Table 3.** Predicted impact of parathyroid hormone on Ca^2+^ transport

			*J*_Ca_, pmol·min^−1^·mm^−1^ per nephron		
	Filtered Load, pmol/min per nephron	%Reabsorption	Net	Paracellular	Transcellular
Base case	37.5	69.3	2.36	2.02	0.34
Reduced expression of Na^+^ transporters*	37.5	49.2	1.68	1.13	0.55
20% decrease in SNGFR	30.0	62.7	1.71	1.50	0.21
20% decrease in SNGFR and reduced expression of Na^+^ transporters*	30.0	47.0	1.28	0.94	0.34

*J*_Ca_, Ca^2+^ flux; SNGFR, single-nephron glomerular filtration rate.

* Basal expression of Na^+^/H^+^ exchanger type 3, Na^+^-PO_4_ cotransporters (NaPi-IIa, NaPi-IIc, and PiT-2), and Na^+^-K^+^-ATPase is lowered by 75%, 75%, and 25%, respectively.

#### Apical Na^+^/H^+^ exchanger type 3-mediated PTH effects.

PTH acts to augment Ca^2+^ reabsorption in the thick ascending limb and below, but in the PT it has been found to reduce *T*_Ca_, at least in dogs (2, 51). This may be explained by the coupling between Na^+^ and Ca^2+^ transport in the PT. PTH is known to inhibit apical Na^+^/H^+^ exchanger type 3 (NHE3), Na^+^-PO_4_ cotransporters, and the basolateral Na^+^-K^+^-ATPase pump (27); the resulting decrease in Na^+^ and water reabsorption in the PT likely reduces Ca^2+^ transport as well. To test this hypothesis, we examined the effects of a 30% decrease in PT Na^+^ reabsorption (as observed in Ref. 2) on *T*_Ca_ at constant SNGFR. Reducing Na^+^ entry into the PT cell lowers intracellular Na^+^ concentration ([Na^+^]), thereby augmenting basolateral Na^+^ secretion via the Na^+^-dependent Cl^−^/$HCO_{3}^{-}$ exchanger NDCBE; a decrease in Na^+^-K^+^-ATPase activity partly counterbalances these effects. Moreover, as transcellular Na^+^ transport decreases, less water is reabsorbed, so that luminal flow decreases less rapidly; the higher microvillous torque then recruits more transporters to the membrane, which conversely enhances transcellular transport. In the following simulations, Na^+^ reabsorption was reduced by 30%, while intracellular [Na^+^] was maintained at >10 mM, by lowering the basal (without torque-mediated effects) expression of NHE3, Na^+^-PO_4_ transporters, and Na^+^-K^+^-ATPase by 75%, 75%, and 25%, respectively. Owing to the compensatory and torque-modulated effects described above, the Na^+^ fluxes across NHE3, Na^+^-PO_4_ transporters, and Na^+^-K^+^-ATPase were equal to 79%, 57%, and 92%, respectively, of their base-case values.

As summarized in Table 3, the 30% decrease in transepithelial Na^+^ transport is predicted to reduce *T*_Ca_ also by 30%, in accordance with experimental observations (2). As water reabsorption diminishes, luminal [Ca^2+^] is lowered, and both convection and diffusion of Ca^2+^ across TJs decrease. The 44% reduction in the paracellular *J*_Ca_ is, however, partially counterbalanced by a 62% increase in the transcellular *J*_Ca_, stemming from a more negative electric potential within the cell cytosol (not shown). Overall, these results indicate that PTH indeed reduces *T*_Ca_ indirectly, via its effects on Na^+^ transport.

PTH is also known to reduce glomerular filtration rate (GFR), perhaps via tubuloglomerular feedback (27). Thus, in the next set of simulations, we both lowered SNGFR by 20% and reduced the basal expression of NHE3, apical Na^+^-PO_4_ transporters, and Na^+^-K^+^-ATPase by 75%, 75%, and 25%, respectively. As shown in Table 3, the effects of decreasing SNGFR and Na^+^ transporter activity on Ca^2+^ transport are almost additive; fractional Ca^2+^ reabsorption is computed as 47.0% vs. 69.3% in the base case.

#### Impact of paracellular Ca^2+^ permeability.

**Fig. 6.**
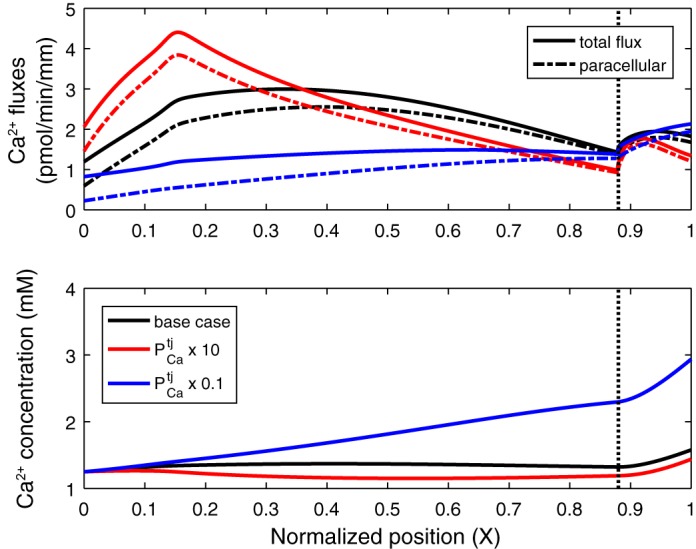
Impact of the permeability of the tight junction to Ca^2+^ ($P_{\text{Ca}}^{\text{tj}}$) on Ca^2+^ fluxes and concentrations along the proximal tubule. *Top*: total and paracellular Ca^2+^ fluxes. *Bottom*: luminal Ca^2+^ concentration. $P_{\text{Ca}}^{\text{tj}}$ is set to its baseline value (base case), multiplied by 10 (× 10), or divided by 10 (× 0.1). Vertical line denotes the boundary between the cortex and the medulla.

There is some evidence that PTH may also decrease paracellular permeability in the PT (23, 31). To our knowledge, the specific effects of PTH on permeability of the TJ to Ca^2+^ ($P_{\text{Ca}}^{\text{tj}}$) have not been investigated. Our model suggests that large reductions in $P_{\text{Ca}}^{\text{tj}}$ may have a substantial effect on overall Ca^2+^ reabsorption, in spite of counteracting effects (Fig. 6). Decreasing $P_{\text{Ca}}^{\text{tj}}$ lowers paracellular *T*_Ca_, thereby raising luminal [Ca^2+^] and, thus, the driving force for transcellular transport; increases in transcellular *T*_Ca_ are limited, however (Table 2). Moreover, if $P_{\text{Ca}}^{\text{tj}}$ is diminished by a factor of ≤5, paracellular *J*_Ca_ becomes higher than in the base case toward the end of the PT, where the effects of the larger [Ca^2+^] gradient (namely, the greater driving force) are sufficient to overcome the effects of the permeability reduction. Nevertheless, overall *T*_Ca_ decreases by >10% if $P_{\text{Ca}}^{\text{tj}}$ is reduced by a factor of >2 (Table 2).

Increasing $P_{\text{Ca}}^{\text{tj}}$ elicits opposite effects, namely, a reduction in luminal [Ca^2+^] and, therefore, in transcellular *T*_Ca_. Furthermore, paracellular *J*_Ca_ becomes lower than in the base case beyond a certain point along the PT (Fig. 6), owing to the reduced lumen-to-interstitium [Ca^2+^] gradient. These compensating mechanisms substantially mitigate the effects of even large (e.g., 10-fold) increases in $P_{\text{Ca}}^{\text{tj}}$, as recapitulated in Table 2. Overall, these results suggest that decreasing and increasing $P_{\text{Ca}}^{\text{tj}}$ induce asymmetric responses.

#### Ca^2+^-sensing mechanisms.

Several studies have suggested that the Ca^2+^-sensing-receptor (CaSR) or CaSR-like molecules may be expressed in the PT (6, 22, 43), but the impact of the CaSR on Ca^2+^ transport in that segment has not been examined and is a subject of debate (32). Capasso et al. demonstrated that treating PT segments with a calcimimetic agent activates NHE-mediated H^+^ extrusion (13). Together, their results suggest that Ca^2+^-sensing mechanisms increase NHE-mediated Na^+^ reabsorption, which in turn enhances fluid reabsorption. Thus these mechanisms and PTH appear to exert opposite effects, as also suggested by other studies (6). To examine the impact of Ca^2+^ sensing on *T*_Ca_, we increased the basal (without torque-modulated effects) expression of NHE3 by 40%. Expression of Na^+^-K^+^-ATPase was raised concomitantly, albeit to a lesser extent (10%), so as to maintain intracellular [Na^+^] below 25 mM. The basal expression of basolateral Na^+^-$HCO_{3}^{-}$ cotransporters was also augmented (by 40%) to prevent large fluctuations in intracellular volume: adjustment of Na^+^-$HCO_{3}^{-}$ cotransport is one of the mechanisms underlying cell volume regulation in the PT (16, 59, 61).

Augmenting the Na^+^ flux across NHE3 raises intracellular [Na^+^], which then decreases Na^+^ entry via Na^+^-PO_4_ cotransporters. It also increases water reabsorption, thus reducing the luminal flow and microvillous torque, such that fewer transporters are recruited to the membrane. Specifically, upregulating Na^+^ transporter activity as described above is predicted to raise fractional Na^+^ reabsorption from 70.1% to 73.5%, which in turn elevates fractional Ca^2+^ reabsorption from 69.3% to 72.8% (Table 2). Together, our results suggest that variations in Na^+^ reabsorption induce nearly identical changes in *T*_Ca_.

#### Impact of acid-base status.

Acetazolamide, a commonly prescribed inhibitor of carbonic anhydrase (CA), raises urinary Ca^2+^ excretion (3, 38). To investigate the impact of acetazolamide in the PT specifically, we reduced the rates of CO_2_-H_2_CO_3_ interconversion in the PT lumen by a factor of 10^−4^ (to uncatalyzed values). As previously described (62), luminal CA inhibition induces PT diuresis, because it abolishes the $HCO_{3}^{-}$ and Cl^−^ gradients that normally drive paracellular water reabsorption. The present model predicts that Na^+^ reabsorption is then reduced by 20%, across both transcellular and paracellular pathways, as apical H^+^ recycling via NHE3 is significantly impaired and the lumen-to-interstitium [Na^+^] gradient is reduced. Ca^2+^ reabsorption is predicted to similarly decrease by 23% (Table 2); an identical 23% acetazolamide-induced decrease was observed in the dog PT (8).

Acetazolamide may also indirectly inhibit the basolateral Na^+^-HCO_3_ transporter NBC1 (50), mutations of which are associated with proximal renal tubular acidosis. Our model suggests that NBC1 inhibition reduces transcellular Na^+^ reabsorption, subsequently diminishing water reabsorption and paracellular Na^+^ fluxes, as well as Ca^2+^ reabsorption. An isolated 90% decrease in NBC1 expression is predicted to reduce *T*_Ca_ from 26.0 to 19.7 pmol/min (Table 2), a 24% decrease. Luminal pH at the PT outlet is computed as 7.34 vs. 7.28 in the base case.

#### Effects of SGLT2 inhibitors.

Inhibitors of SGLT2 are increasingly used to treat diabetes, but they have been linked to bone loss or increased risk of fracture, possibly as a result of altered Ca^2+^ and PO_4_ metabolism (1). SLGT2 inhibition increases urinary Ca^2+^ excretion in rats and mice (33, 35), but whether this is due to a direct effect on proximal Ca^2+^ reabsorption is unknown. We examined the effects of blocking SGLT2 on Ca^2+^ reabsorption in the PT under normoglycemia. SGLT2 inhibition elicits glucose-induced osmotic diuresis, which subsequently reduces the lumen-to-interstitium [Ca^2+^] gradient and, therefore, paracellular Ca^2+^ transport. On the other hand, the higher luminal flow increases microvillous torque, thereby upregulating the membrane abundance of transport proteins and enhancing transcellular Ca^2+^ transport. As a net result, the computed *T*_Ca_ decreases by 12% (Table 2), fractional Ca^2+^ reabsorption decreases by 8.5% (from 69.3% to 60.8%), and delivery of Ca^2+^ to the loop of Henle increases from 11.5 to 14.7 pmol/min per nephron.

By significantly reducing proximal reabsorption, SGLT2 blockers activate tubuloglomerular feedback, which in turn reduces SNGFR. Chronic SGLT2 blockade was found to lower GFR by 15% in diabetic rats (54). When we simulated a 15% decrease in SNGFR in combination with a 100% inhibition of SGLT2, fractional Ca^2+^ reabsorption decreased even further (to 56.6%), and the distal delivery of Ca^2+^ remained higher than in the base case (13.8 pmol/min per nephron). Together, these simulations suggest that SGLT2 blockers may significantly affect renal handling of Ca^2+^ (see below).

#### PO_4_ reabsorption.

The baseline expression of NaPi-IIa, NaPi-IIc, and PiT-2 was chosen so that NaPi-IIa mediates 70% of PO_4_ reabsorption in the PT (27), with the remainder arbitrarily divided equally between NaPi-IIc and PiT-2. In the base case, the model predicts that the PT reabsorbs 79.2% of the filtered load of PO_4_, entirely across transcellular pathways. The tubular fluid-to-glomerular filtrate PO_4_ concentration ratio [(TF/GF)_PO4_] is <1.0 and is comparable to micropuncture measurements in rats and dogs (4, 63). Specifically, the computed value of (TF/GF)_PO4_ decreases from 1.0 to 0.53 along the S1–S2 segment and increases to 0.86 in the S3 segment due to the interstitial medullary concentration gradient.

**Fig. 7.**
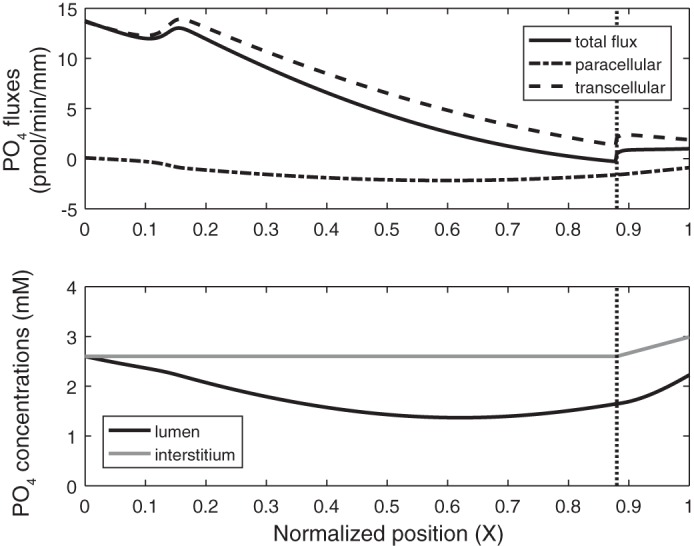
Base-case PO_4_ fluxes and concentrations along the proximal tubule. *Top*: total, paracellular, and transcellular PO_4_ fluxes. *Bottom*: PO_4_ concentration in the lumen and interstitium. Vertical line denotes the boundary between the cortex and the medulla.

If it is assumed that the TJ permeability to PO_4_ equals 4 × 10^−5^ cm/s (62), the model predicts significant PO_4_ backleak across the TJ (Fig. 7). Net PO_4_ reabsorption (*T*_PO4_) is 61.8 pmol/min, with 78.5 pmol/min reabsorbed via transcellular routes and 16.7 pmol/min secreted via paracellular routes. The average PO_4_ flux (*J*_PO4_) is computed as 5.6 pmol·min^−1^·mm^−1^.

The contribution of NaPi-IIa to PO_4_ reabsorption in mice is thought to be ~70%, based on gene knockout studies (7, 46, 53). To assess the importance of compensatory mechanisms, we simulated the effects of inhibiting each type of Na^+^-PO_4_ transporter in turn. Results are summarized in Table 4. Full inhibition of NaPi-IIa is predicted to reduce net PO_4_ reabsorption by 26% (to 45.7 pmol/min) and net Na^+^ reabsorption by 3%. The lower PO_4_ transport rate means that (TF/GF)_PO4_ remains above 1.0, backleak across the TJ into the lumen is abolished, and the paracellular pathway mediates PO_4_ reabsorption instead of secretion; it is predicted to represent 16% of the total flux. The contribution of NaPi-IIc to *T*_PO4_ then exceeds that of PiT-2 (Table 4), likely because NaPi-IIc carries ${\text{HPO}}_{4}^{2-}$ (as does NaPi-IIa), which is more abundant than ${\text{H}}_{\text{2}}{\text{PO}}_{\text{4}}^{-}$, the species carried by PiT-2.

**Table 4.** Predicted PO_4_ fluxes following transporter inhibition

	*J*_PO4_, pmol·min^−1^·mm^−1^ per nephron					
	Net	Paracellular	Transcellular	Across NaPi-IIa	Across NaPi-IIc	Across PiT-2
Base case	5.62	−1.52	7.14	4.98 (69.7%)	1.05 (14.7%)	1.11 (15.5%)
100% inhibition of NaPi-IIa	4.16	+0.65	3.51	0	1.97 (56.2%)	1.54 (43.8%)
100% inhibition of NaPi-IIc	5.38	−1.04	6.42	5.24 (81.5%)	0	1.18 (18.5%)
100% inhibition of PiT-2	5.38	−1.06	6.44	5.23 (81.2%)	1.21 (18.8%)	0

*J*_PO4_, flux of PO_4_ (${\text{HPO}}_{4}^{2-}$ + ${\text{H}}_{\text{2}}{\text{PO}}_{\text{4}}^{-}$); NaPi-IIa, NaPi-IIc, and PiT-2, Na^+^-PO_4_ cotransporters.

When either NaPi-IIc or PiT-2 is fully inhibited, the activity of NaPi-IIa increases by ~5% in compensation. The lumen-to-interstitium PO_4_ concentration ([PO_4_]) gradient still favors paracellular PO_4_ secretion, as in the base case, but the PO_4_ flux across the TJ is nevertheless reduced. As a result of both effects, net *T*_PO4_ is predicted to decrease only slightly, by 4% (Table 4). Net Na^+^ reabsorption diminishes by <1%.

Note that as a baseline, the model assumes that NaPi-IIc and PiT-2 are only expressed in the convoluted PT (42). When we assumed a uniform distribution of NaPi-IIc and PiT-2 along the full PT, the computed *T*_PO4_ increased from 61.8 to 62.6 pmol/min, a 1.3% difference.

#### Effects of PTH on PO_4_ transport.

PTH decreases PO_4_ reabsorption in the PT in a direct manner, by reducing the membrane abundance of NaPi-IIa, NaPi-IIc, and PiT-2 via different mechanisms (10, 24, 37, 42, 47). PTH stimulates the internalization of NaPi-IIa by activating PKA and PKC (10), two kinases that are also involved in NHE3 inhibition (25, 27). We surmised that PTH may also affect *T*_PO4_ indirectly, via its inhibitory action on NHE3. We examined each of these effects, first separately and then simultaneously.

Shown in Table 5 are the computed average PO_4_ fluxes for different degrees of Na^+^-PO_4_ cotransporter inhibition. As expected, PO_4_ reabsorption is predicted to decrease significantly with increasing inhibition; 90% inhibition of all Na^+^-PO_4_ transporters reduces *T*_PO4_ from 61.8 to 36.1 pmol/min. Conversely, NHE3 inhibition by itself is predicted to enhance *T*_PO4_, because it lowers intracellular [Na^+^], thereby stimulating the activity of Na^+^-PO_4_ transporters. This means that the direct effect of PTH on PO_4_ transporter abundance and its indirect effect on PO_4_ transporter activity counteract each other. Consequently, when the abundance of apical PO_4_ transporters is reduced by ≤75%, the predicted *T*_PO4_ is higher when NHE3 inhibition is taken into account than when it is not (Table 5).

**Table 5.** Predicted impact of parathyroid hormone on PO_4_ transport

			*J*_PO4_, pmol·min^−1^·mm^−1^ per nephron		
	Filtered Load, pmol/min per nephron	%Reabsorption	Net	Transcellular	Paracellular
Base case	78.0	79.2	5.62	7.14	−1.52
75% inhibition of Na^+^-PO_4_ transporters	78.0	54.7	3.88	2.69	+1.19
90% inhibition of Na^+^-PO_4_ cotransporters	78.0	46.2	3.27	1.26	+2.01
NHE3 inhibition*	78.0	94.8	6.73	9.92	−3.19
NHE3 inhibition* and 75% inhibition of Na^+^-PO_4_ cotransporters	78.0	55.5	3.93	4.16	−0.23
NHE3 inhibition* and 90% inhibition of Na^+^-PO_4_ cotransporters	78.0	38.6	2.73	1.97	+0.76
20% reduction in SNGFR	62.4	67.1	3.81	4.47	−0.66
20% reduction in SNGFR, NHE3 inhibition* and 75% inhibition of Na^+^-PO_4_ cotransporters	62.4	47.7	2.70	2.59	+0.11
20% reduction in SNGFR, NHE3 inhibition* and 90% inhibition of Na^+^-PO_4_ cotransporters	62.4	35.8	2.03	1.22	+0.81

*J*_PO4_, flux of PO_4_ (${\text{HPO}}_{4}^{2-}$+ ${\text{H}}_{\text{2}}{\text{PO}}_{\text{4}}^{-}$); NHE3, Na^+^/H^+^ exchanger type 3; SNGFR, single-nephron glomerular filtration rate. Fractional inhibition of Na^+^-PO_4_ cotransporters NaPi-IIa, NaPi-IIc, and PiT-2 is taken to be the same.

* Parathyroid hormone is assumed to reduce basal expression of NHE3 and Na^+^-K^+^-ATPase by 75% and 25%, respectively.

Interestingly, the model predicts that when the abundance of apical PO_4_ transporters is reduced by ≥80%, the predicted *T*_PO4_ is lower when NHE3 inhibition is taken into account than when it is not (Table 5). This occurs because, in the latter case (i.e., no indirect, counterbalancing effects via NHE3), transcellular *J*_PO4_ is so small that luminal [PO_4_] increases steeply along the PT, which strongly stimulates PO_4_ reabsorption across the paracellular route. In the former case (i.e., in the presence of counterbalancing effects via NHE3), transcellular *J*_PO4_ remains higher, and paracellular PO_4_ reabsorption is significantly lower (Table 5).

As described above, PTH also reduces GFR. By itself, a 20% decrease in SNGFR is predicted to reduce fractional PO_4_ reabsorption by 12% (from 79.2% to 67.1%). When combined with PTH-mediated inhibition of Na^+^-PO_4_ transporters and NHE3, fractional PO_4_ reabsorption is further reduced (Table 5).

## DISCUSSION

### 

#### Determinants of Ca^2+^ transport in the PT.

The main objective of this study was to elucidate the physical mechanisms underlying Ca^2+^ reabsorption and its regulation in the PT. We expanded a previously published model of transport across the PT, which did not account for Ca^2+^ (26). Our model suggests that the lumen-to-interstitium [Ca^2+^] gradient, which results from water reabsorption, is the main force driving Ca^2+^ transport across the PT epithelium. The transepithelial electric potential difference contributes to the Ca^2+^ flux to a lesser extent, and only in the distal PT, where it is lumen-positive. When we set the valence of Ca^2+^ to zero in our simulations, the computed *T*_Ca_ decreased by only 5% (from 26.0 to 24.8 pmol/min). If it is assumed that the reflection coefficient of the TJ to Ca^2+^ is equal to 0.89 (41), the convective *J*_Ca_ across the TJ is predicted to be approximately seven times lower than the electrodiffusive *J*_Ca_ (Table 2). As previously suggested (17), our model indicates that Ca^2+^ reabsorption in the PT is dominated by passive diffusion.

The nature and contribution of transcellular Ca^2+^ fluxes in the PT are poorly understood. Apical L-type Ca^2+^ channels were identified in cultured rabbit PT cells (65), and colocalization of transient receptor potential channel 1 and aquaporin 1 was observed in rat kidneys (21), but the specific Ca^2+^ molecular transporters in rat PT cells remain to be determined. Interestingly, our results suggest that enhancing transcellular Ca^2+^ transport has a small impact on overall *T*_Ca_, because it induces a counteracting decrease in paracellular Ca^2+^ transport (by reducing the lumen-to-interstitium [Ca^2+^] gradient). Conversely, reducing transcellular Ca^2+^ transport elicits a compensatory increase in paracellular Ca^2+^ transport, such that the overall *T*_Ca_ also does not vary much (Table 2). Hence, it may be difficult to parse the contribution of each pathway unless experiments are carefully designed.

Besides the contribution of transcellular *T*_Ca_, other uncertainties include the magnitude of the corticomedullary interstitial [Ca^2+^] gradient in the medulla (which controls Ca^2+^ reabsorption in the S3 segment), the reflection coefficient of the TJ to Ca^2+^, and the permeability of the BM to Ca^2+^. Our results suggest that the latter three parameters have only a moderate impact on *T*_Ca_ (Table 2).

#### Regulation of Ca^2+^ transport.

Whereas PTH augments Ca^2+^ reabsorption in the thick ascending limb and the distal tubule, studies in dogs suggest that it paradoxically reduces *T*_Ca_ in the PT (2, 51). This reduction is thought to result from PTH-mediated inhibition of NHE3, which diminishes transepithelial Na^+^ and Ca^2+^ fluxes (and fractional reabsorption) by the same factor according to our simulations. PTH is also known to affect GFR. Per se, a PTH-induced decrease in GFR reduces the absolute transport rates of Na^+^ and Ca^2+^, but fractional reabsorption is somewhat maintained by flow-dependent (torque-mediated) transport mechanisms. Since the largest proportion (nearly two-thirds) of the filtered load of Ca^2+^ is reabsorbed in the PT, small variations in transepithelial Ca^2+^ transport in the PT may have a considerable impact on final calciuria, even if Ca^2+^ reabsorption increases downstream via tubular cross talk between segments.

In addition, the model predicts that a (putative) inhibitory action of PTH on TJ permeability to Ca^2+^ may also lower *T*_Ca_ substantially (Table 2); indeed, large decreases in paracellular *J*_Ca_ cannot be compensated for by comparable increases in transcellular Ca^2+^ transport, owing to its limited capacity. The model generally predicts opposite (i.e., counteracting) changes in paracellular and transcellular Ca^2+^ fluxes (Table 2). One exception is when GFR is varied: in this case, Ca^2+^ transport increases (or decreases) both across and between cells, as described above.

Increasing luminal [Ca^2+^] has a demonstrable impact on water and Na^+^ fluxes in the PT (13), but its effects on *J*_Ca_ have not been examined to our knowledge. If it is assumed that Ca^2+^-sensing mechanisms exert only indirect, NHE3-mediated effects on Ca^2+^ transport in that segment, they are predicted to affect Na^+^ and Ca^+^ reabsorption in the same proportion. The model suggests that increases in *J*_Na_ are limited in vivo, owing to flow-dependent transport and the tight coupling between water and Na^+^ transport; thus, Ca^2+^-sensing-induced increases in *J*_Ca_ may be similarly restricted.

The mechanisms by which acetazolamide augments urinary Ca^2+^ excretion remain to be fully characterized. The present study suggests that acetazolamide-induced inhibition of CA in the PT lumen decreases transcellular Na^+^ reabsorption, which in turn lowers paracellular Ca^2+^ fluxes. Our results thus support the hypothesis that acetazolamide-induced calciuria at least partly stems from reduced Ca^2+^ reabsorption in the PT (3).

SLGT2 inhibitors, which are increasingly used to treat diabetes (55), are associated with disturbances in bone metabolism, higher plasma PO_4_ levels, and elevated urinary Ca^2+^ excretion (1, 33, 35). In particular, the mechanisms underlying hypercalciuria remain to be elucidated. Our model predicts that, by itself, blocking SGLT2 in the PT reduces Ca^2+^ reabsorption by 12% in that segment, thereby lowering fractional Ca^2+^ reabsorption from 69.3% to 60.8%. SGLT2 inhibition is also known to decrease SNGFR via tubuloglomerular feedback (54). Even when SNGFR (i.e., the filtered load) is concomitantly reduced by 15–25%, Ca^2+^ delivery to the loop of Henle remains 15–20% higher than in the base case, according to our simulations. This significant increase in Ca^2+^ delivery may not be fully compensated for downstream, given the limited Ca^2+^ transport capacity of distal segments. In other words, our results suggest that the effects of SGLT2 inhibition on Ca^2+^ transport in the PT may contribute to SGLT2 blocker-induced hypercalciuria and bone disease.

In addition, it has been postulated that the increase in plasma [PO_4_] induced by SGLT2 inhibition may stem from increased tubular PO_4_ reabsorption (52). However, our model suggests that *T*_PO4_ may not increase if SNGFR is reduced. Per se, blocking SGLT2 is predicted to raise *T*_PO4_ (by 5.8%) via two mechanisms: not only is the activity of Na-PO_4_ cotransporters stimulated by the decrease in intracellular [Na^+^], but their membrane abundance (and that of other transcellular transporters) is also upregulated in response to the higher luminal flow. Nevertheless, even a small (5%) SGLT2 inhibition-induced decrease in SNGFR more than counterbalances these effects and lowers *T*_PO4_ below its baseline value. If it is assumed that blocking SGLT2 lowers SNGFR by 15% (54), the computed *T*_PO4_ is 20% lower than in the absence of SGLT2 inhibitors.

#### Impact of claudin-2 deletion.

Fractional Ca^2+^ excretion (FE_Ca_) is increased threefold, from 0.13 to 0.40% (40), in claudin-2 knockout mice, likely as a result of impaired Ca^2+^ reabsorption in the PT (19). Claudin-2 is the main cation- and water-permeable channel in the PT (19, 40, 44); its Ca^2+^-to-Na^+^ permeability ratio has been estimated as 1:4 (64). In PT segments specifically, Na^+^, Cl^−^, and water reabsorption is reduced by 20–40% following claudin-2 gene deletion (40, 45). Whether the FE_Ca_ increase stems from altered claudin-2-dependent paracellular Ca^2+^ transport in the PT or is only an indirect consequence of impaired paracellular Na^+^ and water reabsorption remains to be ascertained. To shed light on this question, we simulated a 90% decrease in the paracellular permeability of PT TJs to Na^+^ and water, with or without a concomitant 90% decrease in $P_{\text{Ca}}^{\text{tj}}$.

A 10-fold decrease in the paracellular permeability to Na^+^ and water is predicted to lower their reabsorption by 25% and 24%, respectively. By itself, this reduces *T*_Ca_ from 26.0 to 18.3 pmol/min and fractional Ca^2+^ reabsorption from 69.3% to 48.7%. If $P_{\text{Ca}}^{\text{tj}}$ is reduced by 90% in tandem, the computed fractional Ca^2+^ reabsorption decreases further, to 28.8%; in other words, the load delivered to the thick ascending limb is then 2.3 times higher than under normal conditions. Under these circumstances, it seems unlikely that Ca^2+^ reabsorption mechanisms downstream from the PT (namely, passive reabsorption in the thick ascending limb and active transport in the distal convoluted and connecting tubules) can be sufficiently upregulated to maintain urinary Ca^2+^ excretion at ∼1%. This suggests that the higher FE_Ca_ observed in claudin-2 KO mice might only be an indirect effect, the consequence of reduced Na^+^ and water reabsorption in the PT. A model of transport along the entire nephron would help fully elucidate this question. Experiments designed to block Ca^2+^ reabsorption specifically in the thick ascending limb and/or the distal convoluted tubule of claudin-2 knockout mice would also yield a better understanding of the relative contribution of these segments in compensating for the loss of Ca^2+^ in the PT due to the absence of claudin-2.

#### Determinants of PO_4_ transport.

The model was also expanded to account for the specific stoichiometry of each apical Na^+^-PO_4_ transporter in the PT. In mice, but not in humans, the contribution of NaPi-IIa predominates (9, 27), and we assumed in the base case that NaPi-IIa mediates 70% of *T*_PO4_ vs. 15% each for NaPi-IIc and PiT-2. In addition, permeability of the TJ to ${\text{HPO}}_{4}^{2-}$ and ${\text{H}}_{\text{2}}{\text{PO}}_{\text{4}}^{-}$ was taken as 4.0 × 10^−5^ cm/s (62). With these hypotheses, the model predicts that, under normal conditions, the passive backleak of PO_4_ across the TJ reduces net PO_4_ reabsorption by 21% (Table 4). However, when transcellular PO_4_ transport is substantially impaired, the paracellular PO_4_ flux switches direction and may contribute significantly to PO_4_ reabsorption (Tables 4 and 5). Moreover, when transport via NaPi-IIa is fully blocked, NaPi-IIc flux increases significantly more than PiT-2 flux. Together, these results suggest that it may be difficult to extrapolate measurements in knockout animal models to quantify the contribution of each Na^+^-PO_4_ transporter.

PTH is known to reduce PO_4_ reabsorption in the PT by lowering the membrane expression of NaPi-IIa, NaPi-IIc, and PiT-2 (27). PTH may also impact *T*_PO4_ indirectly by inhibiting NHE3. According to our simulations, inhibition of NHE3 per se stimulates the activity of Na^+^-PO_4_ transporters, suggesting that the direct and indirect effects of PTH on *T*_PO4_ counteract each other. Moreover, when transcellular PO_4_ reabsorption is severely reduced, paracellular PO_4_ reabsorption may increase very significantly in compensation. PTH-induced decreases in GFR may also contribute to lowering *T*_PO4_ (Table 5).

#### Possible model extensions.

Since the molecular transporters that mediate transcellular Ca^2+^ reabsorption in the PT remain to be identified, our model assumes that the transcellular *J*_Ca_ is solely driven by the Ca^2+^ electrochemical potential gradient: this assumption is valid if Ca^2+^ entry into the cell occurs via a Ca^2+^ channel, but not if it is mediated by a cotransporter. Further experimental studies are needed to clarify this. Additionally, the current model could be expanded in several ways. It does not account for the binding between Ca^2+^ and ${\text{HPO}}_{4}^{2-}$ or ${\text{H}}_{\text{2}}{\text{PO}}_{\text{4}}^{-}$, the rate of which is pH-dependent. Nor does it include phospho- and calcitropic hormones other than PTH, such as fibroblast growth factor 23 (FGF23) and vitamin D_3_. Vitamin D_3_ enhances the intestinal absorption of both Ca^2+^ and PO_4_, and its synthesis in PT cells is activated by PTH (5) and, conversely, inhibited by FGF23 (48). However, whether vitamin D_3_ directly modulates Ca^2+^ and PO_4_ fluxes in the PT remains to be ascertained (27). FGF23 is an important regulator of PO_4_ metabolism; in the PT, it reduces PO_4_ reabsorption by decreasing NaPi-IIa and NaPi-IIc expression (20). FGF23 requires α-klotho as a cofactor, which is expressed mainly in the distal tubule and, to a much lower extent, in the PT (29). Thus the actions of FGF23 in the PT may be indirect and may involve a distal-to-proximal tubular feedback mechanism that has yet to be elucidated (36). Finally, this PT model should be linked to our models of Ca^2+^ transport in the distal nephron (12, 15) to yield an integrated understanding of renal Ca^2+^ handling.

In conclusion, we have developed the first model of Ca^2+^ transport in the PT. Our results indicate that Ca^2+^ reabsorption in that segment is principally driven by the lumen-to-interstitium [Ca^2+^] gradient that is generated by water reabsorption. Our model also provides greater insight into the different mechanisms by which the reabsorption of Ca^2+^ and PO_4_ is regulated in that segment.

## GRANTS

O. Bonny is supported by a special program of the Swiss National Foundation (National Centre of Competence in Research, Kidney, Control of Homeostasis) and Swiss National Foundation Grant 310030-163340.

## DISCLOSURES

No conflicts of interest, financial or otherwise, are declared by the authors.

## AUTHOR CONTRIBUTIONS

A.E. and O.B. conceived and designed research; A.E. performed experiments; A.E. analyzed data; A.E. and O.B. interpreted results of experiments; A.E. prepared figures; A.E. drafted manuscript; A.E. and O.B. edited and revised manuscript; A.E. and O.B. approved final version of manuscript.
